# Conservative management of trachea-to-innominate artery transfixion with a guidewire during percutaneous tracheostomy: a case report

**DOI:** 10.1186/s12871-019-0893-5

**Published:** 2019-12-10

**Authors:** Giancarlo Varelli, Roberto Cioni, Sergio Casagli, Rosa Cervelli, Claudia Brusasco, Francesco Forfori, Francesco Corradi

**Affiliations:** 1 Neuroanestesia e rianimazione, Ospedale Nuovo S. Chiara, Pisa, Italy; 2 Division of Interventional Radiology, University of Pisa, Pisa, Italy; 3 Ente Ospedaliero Ospedali Galliera, Anestesia e Rianimazione, Genova, Italy; 4 Department of Surgical, Medical and Molecular Pathology and Critical Care Medicine, University of Pisa, Via Paradisa 2, 56124 Pisa, Italy

**Keywords:** Neck ultrasonography, Vascular complication, Percutaneous tracheostomy, Bronchoscopy, Innominate trunk transfixion

## Abstract

**Background:**

Tracheostomy is a standard procedure in critically ill patients requiring mechanical ventilation or airway protection for extended periods. The main cause of death is haemorrhage, most commonly owing to a trachea-to-innominate artery fistula, usually requiring surgical treatment.

**Case presentation:**

Here we report the case of an 83-yr-old woman with a subarachnoid haemorrhage, who incurred a trachea-to-innominate artery transfixion following percutaneous tracheostomy, successfully and conservatively managed by interventional radiology.

**Conclusions:**

The use of peri-procedural ultrasound examination of the neck can reduce the risk of complications related to vessel anatomical variants. When the tracheostomy is complicated by bleeding, the procedure should be stopped in order to diagnose the vascular iatrogenic injury and to evaluate the best therapeutic approach by a multidisciplinary team.

## Background

Tracheostomy is a standard procedure in critically ill patients requiring mechanical ventilation or airway protection for extended periods. Intraoperative complications (severe desaturation, death, cerebrovascular accidents, pneumothorax, and severe blood loss) have an incidence of 1.4%. Postoperative complications may occur early or late in 5.6 and 7.1% of cases, respectively [1]. The main cause of death is haemorrhage (38%), most commonly owing to a trachea-to-innominate artery fistula, occurring either during or after tracheotomy procedure. This usually requires surgical treatment, [2–4] whereas selected cases are amenable to endovascular treatment by stent placement [3–5]..

## Case presentation

Here we report a case of intra-operative direct injury of the innominate artery caused by percutaneous dilatational tracheostomy, conservatively managed by interventional radiology. The patient was an 83-yr-old woman with a subarachnoid haemorrhage due to the rupture of a right carotid-ophthalmic aneurysm, treated 10 years before by endovascular embolization with coils. The CT scan showed a bi-frontal hematoma, which required the positioning of an external ventricular drain. Five days later, a skilled operator performed percutaneous dilatational tracheostomy under optical-fibre guidance. The neck was thin, with no obvious abnormality, and the trachea was easily palpable. There was no preoperative ultrasound examination of neck, because it is not routinely mandatory in our hospital when there are no obvious neck abnormalities.

**Fig. 1:**
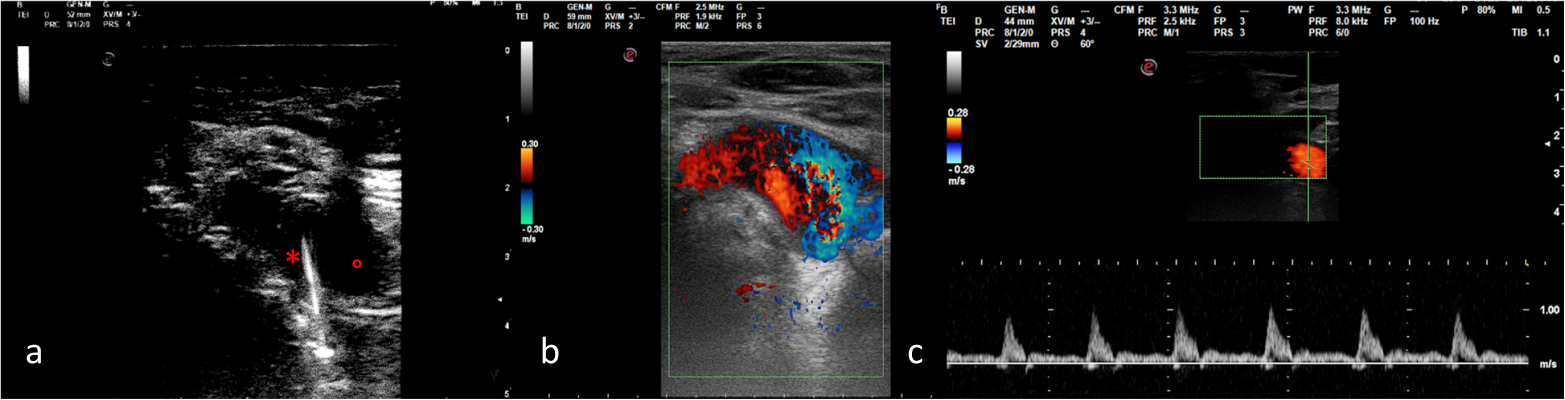
panel **a**, suprasternal notch acoustic window, short axis ultrasound image showing the innominate artery crossed by the guide wire. * guidewire, ° innominate artery; panel **b**, innominate artery color Doppler appearance; panel c, innominate artery pulsed-wave Doppler

**Fig. 2:**
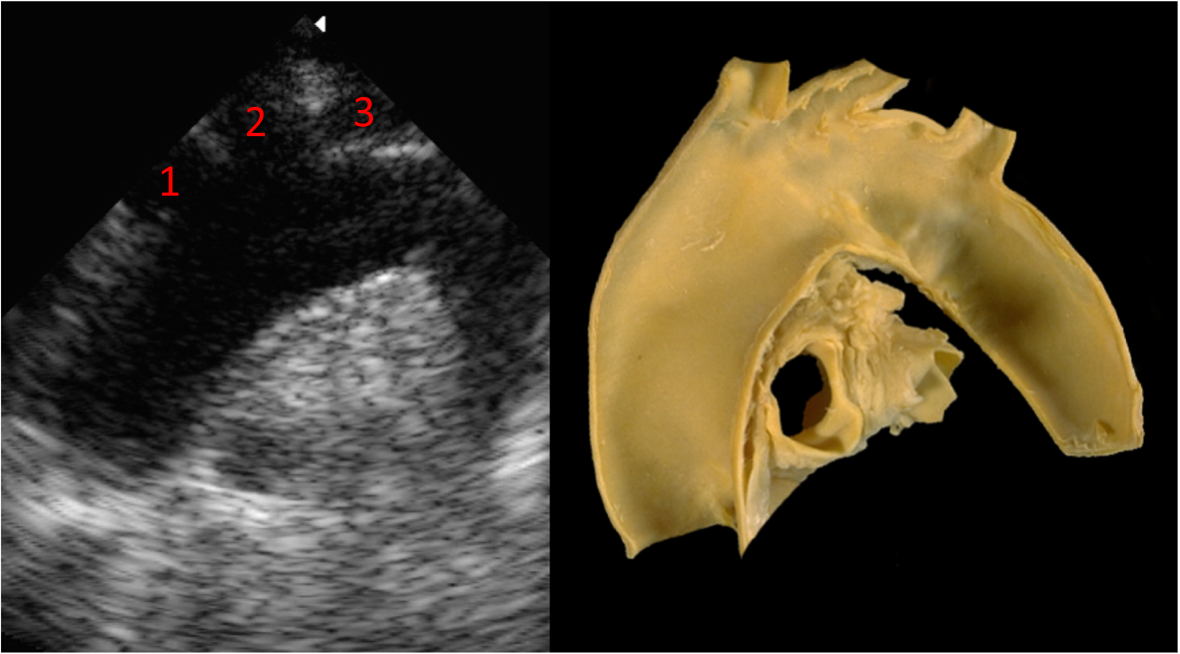
In the picture on le right side an anatomical model of aortic arch and on the left side the corresponding US suprasternal scan. The main trunks originating from the arch are 1) innominate artery 2) lefts carotid artery 3) left subclavian artery

**Fig. 3:**
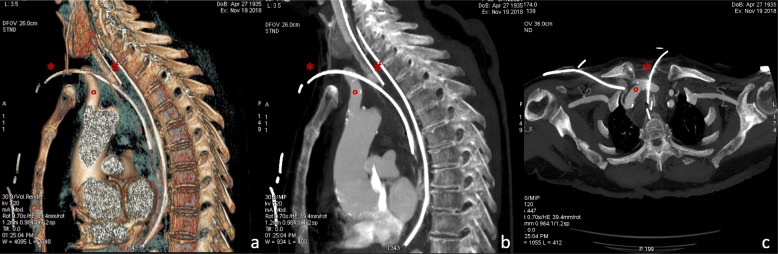
Panel **a**, sagittal view CT 3-dimensional volume rendering; panel **b**, sagittal view CT image; panel **c**, axial view CT image showing the innominate trunk crossed by the guidewire. * guidewire, ° Brachiocefalic trunk, # oro-tracheal tube

The patient was lying supine, deeply sedated, paralyzed and mechanically ventilated to minimize the increase in intracranial pressure during the procedure. An experienced attending intensivist made a small midline transverse incision between second and third tracheal rings. The introducer needle was inserted, during suctioning, into the trachea at first attempt under direct visualization using a fiberoptic bronchoscope and its correct location within the trachea confirmed by aspiration of air. The guidewire was easily passed through the needle with no evidence of bleeding. Upon needle removal, however, slight bleeding was observed on the skin and bronchoscopy showed the presence of intra-tracheal bleeding around the guidewire. The patient remained normotensive and non-anaemic but bleeding, albeit slight, was persistent. Thanks to the presence in the ward of a physician skilled in ultrasonography, the neck was scanned to identify the source of bleeding. B-mode imaging showed the guidewire crossing the lumen of a pulsatile vessel, characterized by an arterial pattern, referable to the innominate artery just before its bifurcation into left internal carotid and subclavian artery. (Fig. 1-2) The procedure was then suspended without widening the puncture channel with a punch dilator. After slow deflation of the tip cuff under bronchoscopic control, the oro-tracheal tube was gradually withdrawn to identify the source of bleeding. The cuff was then hyperinflated just beyond the guidewire tip in order to stop bleeding. The oro-tracheal tube was left in the trachea to secure the airway in case of sudden bleeding, which might have hampered visualization of airways for endotracheal tube positioning. Chest computed tomography confirmed the ultrasonographic diagnosis (Fig. 3), also showing the presence of mediastinal hematoma.

**Fig. 4:**
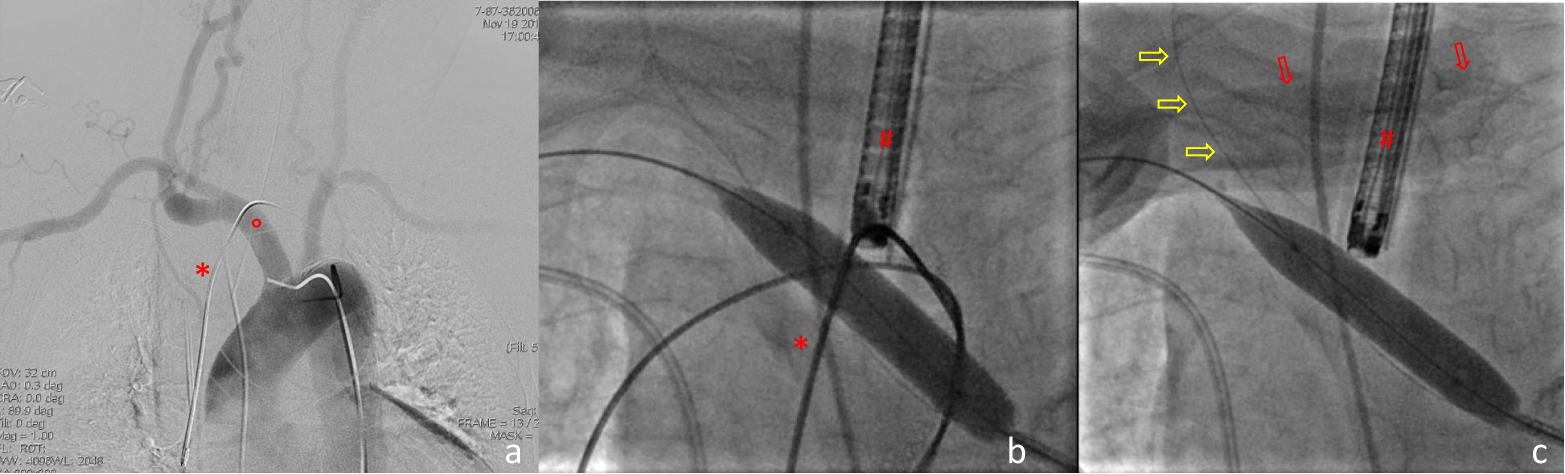
Panel **a**, angiographic procedure showing the catheter placed into the right brachiocephalic artery; panel **b**, intravascular balloon positioned in the injured segment of the brachiocephalic arterial wall; panel **c**, guidewire left in the trachea drawn back and removed. • guidewire, ° Brachiocefalic trunk, # oro-tracheal tube, Red Arrows showing the fingerprint during external manual compression. Yellow arrows showing the guidewire placed into the right carotid artery

Considering the surgical risks, a conservative intravascular approach was chosen with fluoroscopic and bronchoscopic guidance, involving a multidisciplinary team. The use of a covered stent in the innominate artery was excluded as first option, because of the proximity of the lesion to the carotid artery origin and because it would have implied the use of double antiplatelet therapy. After removal of residual clots, a catheter was placed into the right brachiocephalic artery (Fig. 4 panel a) through femoral artery access and a guidewire placed into the right carotid artery (Fig. 4 panel b and c). This step assured the maintenance of communication between right carotid artery and aortic arch during the following interventional procedures. Indeed, due to the close proximity of the carotid artery to the innominate trunk lesion, the guidewire in the carotid artery was necessary to allow the identification of the carotid origin despite the inflated balloon positioned in the innominate trunk. Moreover, the guidewire would have provided a safe access to the carotid artery in case an emergency stent was required due to a damage of the carotid origin during the interventional procedure.

Another guidewire was inserted into the innominate artery and an intravascular balloon positioned in the segment where the brachiocephalic arterial wall was injured (Fig. 4 panel b). After slight balloon deflation, the guidewire left in the trachea was pulled back and removed. Immediately after, the pressure of the balloon was increased for haemostatic purpose and an external manual compression was applied to the surgical wound (Fig. 4 panel c). Five minutes later, the balloon was deflated under optical-fibre visual control. There was no evidence of endotracheal bleeding and this was eventually confirmed by angiographic examination. A surgical tracheostomy was done one week later by an otorhinolaryngologist, without complications.

## Discussion

Hemorrhage is one of the fearsome complications of tracheostomy. Trachea-to-innominate artery fistula is a rare but often fatal complication of tracheostomy. Without intervention, mortality is nearly 100% because of acute massive tracheal haemorrhage. Although the survival rate is extremely low, survival is possible only with immediate intervention.

The vulnerability of major vessels during percutaneous tracheostomy insertion is due to their high variability. Magnetic resonance imaging in normal subjects demonstrated that the innominate artery may originate from the aortic arch left to the midline and therefrom cross the midline anterior to the trachea, above the upper edge of sternal manubrium and close to the site available for percutaneous tracheostomy [6]. In imaging studies the upper edge of the innominate artery was located above the suprasternal notch in 26.4% of patients and across the anterior midline of the trachea 2 cm above the suprasternal notch in 2.2% of all cases [6, 7].

It is perhaps surprising that major vessels are infrequently damaged during percutaneous tracheostomy, given that puncture of the trachea and dilation of the pre-tracheal tissues is essentially blind. Fibre-optic bronchoscopy is unlikely to prevent or reduce these complications, for pre-tracheal vessels are not visualised. The palpation of neck cannot exclude vascular aberrations and the easy finding of landmarks may give deceptive security.

While bronchoscopy is routinely used during percutaneous tracheostomy in most hospitals, ultrasonographic neck examination is not, though it is suitable to identify major vascular structures at risk. Moreover, ultrasonography can identify tracheal midline, levels of cartilaginous rings and overlying or vulnerable adjacent structures, e.g., thyroid. It also allows direct observation of the tracheostomy tube placement into the trachea and prevent its cranial misplacement [8]. In this connection, it is important to note that percutaneous tracheostomy is usually done with extended neck, to bring the trachea to a more anterior position, to eliminate skin folds and make palpation easier, but this brings the brachiocefalic trunk closer to trachea [6]. Thus, correct evaluation of the anatomical relationships of large vessels requires ultrasound examination be done with the patient is positioned with his/her neck extended.

In our hospital, percutaneous dilatational tracheostomy is usually performed with puncture and guidewire insertion first [9], though in other centres it is performed in a different way, i.e., with skin incision, blunt preparation of tracheal wall and subsequent puncture of trachea and insertion of guidewire [10]. The latter method, allows exposure of the anterior surface of the trachea and detection of otherwise not predictable vascular variances. In addition to avoiding injury to pre-tracheal vascular structures, it helps guide safe tracheostomy placement by better knowledge of the anatomy of neck. However, haemorrhage is a recognized hazard of any tracheostomy and has been reported to occur in up to 5% of all cases, irrespective of the technique (surgical or percutaneous) used [11].

In view of the high risk of morbidity and mortality linked to trachea-to-innominate artery fistula and the poorly informative clinical examination, it is our opinion that percutaneous dilatational tracheostomy should be interrupted even in case of minimal tracheal bleeding and real-time intra-operative ultrasonograhy of the neck obtained in order to identify the source of bleeding.

Concerning the treatment, immediate management of innominate artery haemorrhage includes securing the airway, preventing aspiration of blood, minimizing blood loss, fluid resuscitation and definitive control of haemorrhage by early confirmation of the bleeding site. When the innominate artery is identified as the source of haemorrhage, adequate access to the vessel usually requires median sternotomy and cardiopulmonary by-pass [12, 13], which was excluded in our patient because it would have required anticoagulation.

The successful management of trachea-to-innominate artery fistula with an endovascular stent graft repair [14] has been previously reported. As in our case the artery was pinched and transfixed by a Seldinger guide but not dilated, we thought that compressing the bleeding site could be successful, thus avoiding the positioning of an endovascular stent that would have required antiplatelet therapy.

Even if the occlusion of the innominate artery could theoretically carry a significant risk of neurological complications [15, 16], the transient interruption of innominate artery is usually associated with a relatively low incidence of neurological sequelae [17–19]. This is because the occlusion of blood flow through the common carotid and subclavian arteries is likely to be compensated by collateral flows [20] from intercostal and internal mammary arteries and intracranial anastomoses between internal carotid and vertebral arteries [21]. Notwithstanding, to minimize this risk, we left a guide inserted in the internal carotid artery to position a stent, in case the hemostasis procedure were unsuccessful, or a shunt tube.

Our patient recovered with no focal neurological deficits. We did not monitor regional cerebrovascular hemoglobin oxygen saturation by near-infrared spectroscopy to confirm the adequacy of collateral circulation during the procedure, even if it could be useful in order to monitor perfusion, which could be resumed temporarily by controlling the anastomotic region with a finger or by using a shunt tube.

In conclusion, we suggest a pre-procedural neck ultrasonography be performed with neck extended for tracheotomy. In case of bleeding during PDT, we suggest not to remove the orotracheal-tube, overinflate the cuff to prevent blood aspiration and take neck ultrasound before surgical procedures.

## Data Availability

All data included in the section of Case Presentation are available from the corresponding author on reasonable request.

## Abbreviation

CT: Computed Tomography
